# Alkaline and acidic soil constraints on iron accumulation by Rice cultivars in relation to several physio-biochemical parameters

**DOI:** 10.1186/s12870-023-04400-x

**Published:** 2023-08-19

**Authors:** Ammara Saleem, Asma Zulfiqar, Muhammad Zafar Saleem, Baber Ali, Muhammad Hamzah Saleem, Shafaqat Ali, Ebru Derelli Tufekci, Ali Rıza Tufekci, Mehdi Rahimi, Reham M. Mostafa

**Affiliations:** 1 Institute of Botany, University of the Punjab Lahore, Lahore, 54590 Pakistan; 2 Centre for Applied Molecular Biology, University of the Punjab Lahore, Lahore, 54590 Pakistan; 3 Department of Plant Sciences, Quaid-i-Azam University, Islamabad, 45320 Pakistan; 4 College of Plant Science and Technology, Huazhong Agricultural University, Wuhan, 430070 China; 5 Department of Environmental Sciences and Engineering, Government College University, Faisalabad, 38040 Pakistan; 6 Department of Biological Sciences and Technology, China Medical University (CMU), Taichung City, 40402 Taiwan; 7 Food and Agriculture Vocational School, Department of Field Crops, Cankiri Karatekin Universitesi, 18100 Cankiri, Turkey; 8 Faculty of Science, Department of Chemistry, Cankiri Karatekin Universitesi, Cankiri18100, Turkey; 9 Department of Biotechnology, Institute of Science and High Technology and Environmental Sciences, Graduate University of Advanced Technology, Kerman, Iran; 10 Department of Botany and Microbiology, Faculty of Science, Benha University, Benha, 13518 Egypt

**Keywords:** Iron biofortification, Iron fertlizer, Soil fertility, Soil pH, Fe accumulation

## Abstract

Agricultural production is severely limited by an iron deficiency. Alkaline soils increase iron deficiency in rice crops, consequently leading to nutrient deficiencies in humans. Adding iron to rice enhances both its elemental composition and the nutritional value it offers humans through the food chain. The purpose of the current pot experiment was to investigate the impact of Fe treatment in alkaline (pH 7.5) and acidic (pH 5.5) soils to introduce iron-rich rice. Iron was applied to the plants in the soil in the form of an aqueous solution of FeSO_4_ with five different concentrations (100, 200, 300, 400, and 500 mM). The results obtained from the current study demonstrated a significant increase in Fe content in *Oryza sativa* with the application of iron in both alkaline and acidic pH soils. Specifically, Basmati-515, one of the rice cultivars tested, exhibited a notable 13% increase in iron total accumulation per plant and an 11% increase in root-to-shoot ratio in acidic soil. In contrast to Basmati-198, which demonstrated maximum response in alkaline soil, Basmati-515 exhibited notable increases in all parameters, including a 31% increase in dry weight, 16% increase in total chlorophyll content, an 11% increase in CAT (catalase) activity, 7% increase in APX (ascorbate peroxidase) activity, 26% increase in POD (peroxidase) activity, and a remarkable 92% increase in SOD (superoxide dismutase) in acidic soil. In alkaline soil, Basmati-198 exhibited respective decreases of 40% and 39% in MDA and H_2_O_2_ content, whereas Basmati-515 demonstrated a more significant decrease of 50% and 67% in MDA and H_2_O_2_ in acidic soil. These results emphasize the potential for targeted soil management strategies to improve iron nutrition and address iron deficiency in agricultural systems. By considering soil conditions, it is possible to enhance iron content and promote its availability in alkaline and acidic soils, ultimately contributing to improved crop nutrition and human health.

## Introduction

Rice is the best species for genetic characterization of Iron (Fe) homeostasis and genetic improvement since it is the most adaptable model for cereals and a food crop with economic importance [1]. Two thirds of people are fed by rice, a basic staple diet. In developing nations, it is essential for ensuring food security [2]. In order to reach Zero Hunger, hidden hunger must be tackled as one of the primary difficulties, especially in African, Asian, and Latin American nations where it affects more than two billion people. In fact, almost three million people die each year from nutritional deficiencies, primarily due to a deficiency of vitamins and minerals [3].

Numerous vital activities, including as development, cognition, the immune system, and maintaining antioxidant activity, depend on micronutrients like Zn, Fe, and Se [4]. Fe plays a critical role in the catalytic activities of numerous enzymes, including those involved in the transport of oxygen, the transfer of electrons, oxi-reduction events, collagen manufacture, and the metabolism of vitamin D [5]. Anemia, which is defined as having inadequate red blood cells, accounts for a significant number of cases [6]. Children and adults with chronic IDA (Iron Deficiency Anemia) experience substantial growth and developmental problems, including delayed growth, which results in weariness and lowers physical and mental function [7].

The fourth most prevalent element in the earth’s crust is iron (Fe), and it is crucial for both chemical and biological activities. Recent decades have seen an increase in interest in environmental chemistry and material sciences about the redox reactivity of diverse forms of Fe [8]. Fe is an essential component of many enzymes and proteins in vital processes that support growth and metabolism in plants, and a lack of it is directly linked to a decline in crop production and quality because it is crucial for plant growth, productivity, and quality. Algae and higher plants’ photosynthetic efficiency is significantly influenced by Fe homeostasis [9]. Fe has an impact on the cycling of nitrogen (N) in soils in both oxic and anoxic conditions. The pH of the soil controls how much Fe is used in the conversion of N. Fe oxides frequently stimulate nitrification activity in soil with low pH, where their impact on soil N transformation activities is dependent on soil pH [10].

The importance of iron in rice accumulation is attributed to its role in the synthesis and functioning of proteins and enzymes involved in the transport and storage of nutrients. Iron is particularly important in the synthesis of iron-containing proteins, such as ferredoxins, which are essential for electron transfer reactions in photosynthesis and respiration. These processes are critical for energy production and metabolism within the plant [11, 12]. Iron deficiency in rice occurs due to poor solubility in flooded soils., Alkaline or high pH, excessive phosphorus, and other nutrients [13, 14].

One of the main environmental factors that hinders plant development and yield production globally is alkaline stress. Alkaline or high pH soils negatively affect iron availability. In such conditions, iron tends to form insoluble compounds, making it difficult for rice plants to take up sufficient iron Additionally, alkaline stress caused oxidative damage in plants that was reflected in greater levels of superoxide radical (O_2_•), hydrogen peroxide (H_2_O_2_), methylglyoxal (MG), and malondialdehyde (MDA) [15]. On alkaline soils, plants may exhibit iron deficiency chlorosis (IDC), which inhibits development and yield [16]. Alkaline (high pH) soils may also inhibit the absorption of other metal micronutrients such as Mn and Zn [17]. The concentration of carbonate (CO_3_^2−^) and bicarbonate (HCO_3_) increases with a rise in the pH of the growth medium, while the solubility of iron decreases as a result of H^+^ being consumed by HCO_3_ [18]. Numerous research offer specific solutions to address this issue and improve the micronutrient content in crops [19, 20].

More than 50% of the world’s population is fed on rice (*O. sativa* L.). One of the most essential staple crops is rice, but polished rice has a low quantity of important micronutrients, various strategies such as biofortification are useful to cope with this problem and increase the concentration of micronutrients in crops [21, 22]. As a result, the major purpose of current research work is to enhance the content of iron in four *O. sativa* “cultivars: Basmati-198, Basmati-515, PK-386, and KSK-133”. It was also determined how different iron treatments alter morphological, physiological, antioxidant defense systems, and iron absorption. For this purpose, a study was directed to investigate the response of *O. sativa* to various concentrations of FeSO_4_ at two pH levels (alkaline and acidic), as well the suitable Fe concentration and pH for normal rice growth and development in iron deficient soil.

## Materials and methods

### Experimental plan

Seeds of rice (*Oryza sativa* L.) were received from the Rice Research Institute, KSK, and the rice varieties used in the present research work were “Basmati-198, Basmati-515, PK-386, and KSK-133”. Two Basmati varieties were selected in present research work According to our earlier research, the iron concentrations of Basmati-515 and Basmati-198, are 22.0 and 14.1 ppm, respectively, whereas PK-386 has 19.0 ppm [23]. Seeds of four rice cultivars surface sterilized by using 0.1% bleaching powder (10 to 20 min), then gently rinsed with deionized water before being planted in plastic pots in a natural environment condition (day temperature: 36 ^o^C and night temperature: 27 ^o^C).

### Soil preparation

The garden soil (silt loam) from the field of the Botanical Garden, University of the Punjab, Lahore (31^o^29’57.78 N latitude and 74^o^17’58.60 E longitude) was collected. The soil samples were air-dried, and their physico-chemical properties are presented (Table 1).

**Table 1:** The physico-chemical properties of soil samples

Properties	Acidic soil	Alkaline soil
Depth (cm)	0–12	0–12
**pH**	**5.5**	**7.5**
Electrical Conductivity (dS/cm)	0.7	0.7
Organic matter (% )	0.19	0.19
Available phosphorus (mg/kg)	2.98	2.43
Available potassium (mg/kg)	66.4	52.4
Available nitrogen (mg/kg)	94	32
Iron (mg/kg)	67	51
Saturation (% )	25%	25%
Texture	Silty loam	Silty loam

Throughout the experiment, the pH of the soil for each treatment used in this study was adjusted to 5.5 for acidic soil and 7.5 for alkaline soil, respectively. In this experiment, a randomized complete block design (RCBD) with six replications was used, and iron sulfate (FeSO_4_) was manually mixed to achieve the five treatment concentrations (100, 200, 300, 400, and 500 mM) along with the control. The control plants were left untreated, without the addition of any specific treatments.

### Morphological parameters

The plants were harvested on July 16, 2022, after 25 days of treatment, for the different morphological and biochemical parameters. To eliminate dirt and waste, the plants from each treatment were first washed with tap water and then with distilled water. After the plants were harvested, morphological measurements including total plant length, shoot fresh weight, root fresh weight, and total dry weight per plant were recorded in alkaline as well as acidic soil.

#### Iron accumulation in plant

Samples of Fe-added rice varieties were collected to assess the Fe concentration in their tissues. Whole plants washed with distilled water after harvesting to remove soil, and either after all these plants kept in cold DCB (dithionite-citrate-bicarbonate) solution for 3 h for iron content determination. Samples were heated to 500 °C for 3 h and then dried at 60 °C. By using concentrated HCl to decompose the ashes, iron was then measured using atomic absorption spectrophotometry [24].

### Estimation of antioxidant enzyme activities

A leaf of fresh plant material weighing 0.2 g was taken and homogenized in phosphate buffer (5 mL) and liquid nitrogen having a pH of neutral. At 4 °C and 12,000 rpm, the homogenate mixture was centrifuged for 20 min, and then the extracts were stored at -20 °C while the supernatants were discarded.

The antioxidant enzymes CAT [25], APX [26], POD [27], SOD [28], GPX [29] and DPPH [30] were measured at absorbance of 550, 290, 420, 405, 460, 460, and 517 nm respectively.

### Estimation of oxidative stress markers

A mixture of 3 mL of sample extract and 1 mL of 0.1% titanium sulphate in 20% (v/v) H_2_SO_4_ was centrifuged at 6000 g for 15 min to determine the H_2_O_2_ content of plant tissues. The yellow colour intensity was measured at 410 nm [31]. According to [32], the quantity of MDA was determined.

## Results

### Effect of Fe on morphology of *O. sativa* cultivars in soil (pH 7.5 and pH 5.5)

**Fig. 1:**
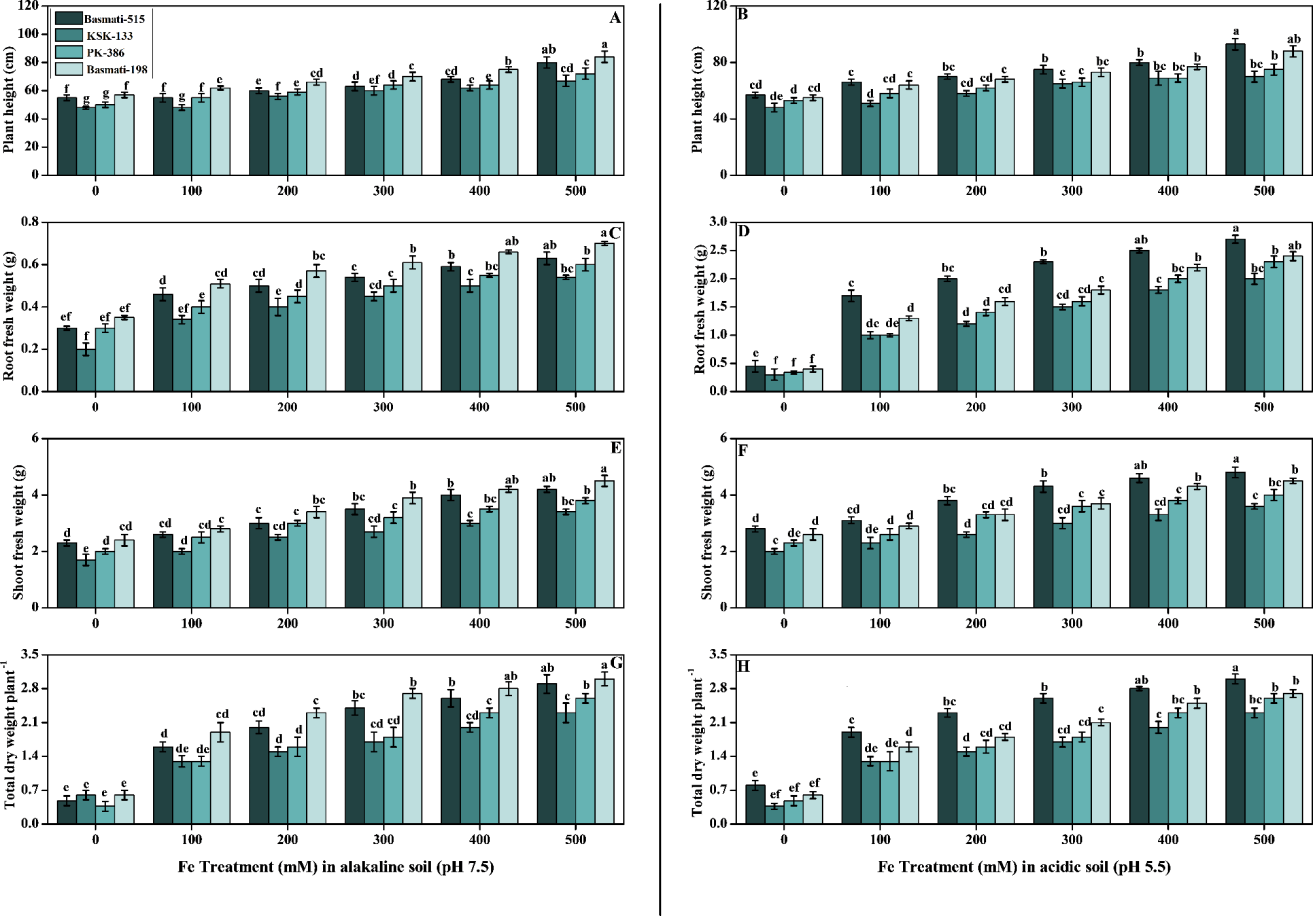
Effect of Fe (0, 100, 200, 300, 400, and 500 mM) on selected *O. sativa* cultivars under alkaline and acidic pH. Plant height (**A, B**), root fresh weight (**C, D**), shoot fresh weight (**E, F**), and total dry weight per plant (**G, H**). According to DMRT, bars with distinct alphabet letters are substantially (p < 0.05) different from one another. All data in the graph are the averages of six replicates (n = 6)

After 25 days of treatment, plants of all *O. sativa* cultivars planted in high doses of Fe amended soils displayed a better plant growth with higher height and weight. Compared to control plants, *O. sativa* cultivars exhibited different growth trends at varying FeSO_4_ concentrations. In Fig. 1 (A, B), displayed the various morphological characteristics of the *O. sativa* cultivars. Findings of the study showed that plant height, root fresh weight, shoot fresh weight, and total dry weight per plant increased by increasing Fe concentrations (100, 200, 300, 400, and 500 mM) than those of control plants. Results from the current study also indicated that at all Fe levels, acidic soil (pH 5.5) produced more significant effects from Fe treatment than alkaline soil with a pH of 7.5. At 500 mM Fe treatments, the height of Basmati-198 and Basmati-515 increased in comparison to control plants by 47% and 45%, respectively (Fig. 1A) in soil having pH alkaline (pH 7.5). Exogenous Fe supply in acidic soil dramatically increased plants height as compared to plants grown in alkaline soil. Basmati-515 in acidic soil showed maximum plant height results that were 63% higher than the control and Basmati-198 in alkaline soil (Fig. 1B). In alkaline soil, Basmati-198 exhibited a substantial increase in root, shoot fresh weight, and dry weight, with respective increments of 94%, 71%, and 491% compared to untreated plants. Additionally, when compared to Basmati-198, Basmati-515 displayed a 285% and 6% increase in root and shoot fresh weight, respectively, and a remarkable 31% increase in total dry weight in acidic soil. These findings underscore the contrasting growth responses between the two cultivars, indicating the superior performance of Basmati-515 in terms of root and shoot fresh weight and total dry weight. In control plants, total dry weight was significantly lower as compared to Fe-treated plants (Fig. 1C- H).

### Effect of Fe on photosynthetic parameters of *O. sativa* cultivars in soil (pH 7.5 and pH 5.5)

**Fig. 2:**
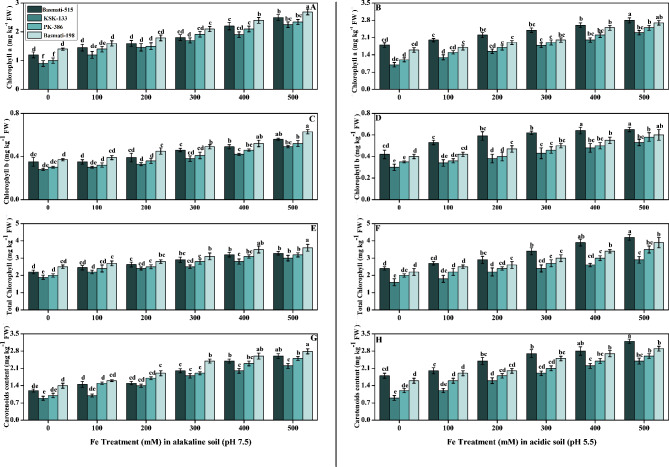
Effect of Fe (0, 100, 200, 300, 400, and 500 mM) on selected *O. sativa* cultivars under alkaline and acidic pH. Chlorophyll a (**A, B**), chlorophyll b (**C, D**), total chlorophyll content (**E, F**), and carotenoids (**G, H**). According to DMRT, bars with distinct alphabet letters are substantially (p < 0.05) different from one another. All data in the graph are the averages of six replicates (n = 6)

The chlorophyll and carotenoids analysis revealed that application of FeSO_4_ enhanced chlorophyll and carotenoids content in *O. sativa*. The results regarding chlorophyll and carotenoids (chl-a, chl-b, and total chlorophyll content) in alkaline and acidic soil under FeSO_4_ treatment are presented in Fig. 2. Chlorophyll-a increased by 85 and 65% in alkaline and acidic soils, respectively, in *O. sativa* cultivar (Basmati-198) in contrast to control. While the highest chl-a was observed in acidic soil, it was observed in Basmati-515 with an average value of 2.8 mg kg^− 1^ FW at the highest concentration 500 mM in comparison to the control with a value of 1.8 mg kg^− 1^ FW (Fig. 2A, B). The chl-b content was significantly increased with the same pattern as in chl-a, but in comparison, the chl-a content was higher at each concentration in all cultivars (Figure C, D). Total chlorophyll content was strongly correlated with chl-a and chl-b content. The results regarding this showed that total chlorophyll content was significantly high in Basmati-515, with values of 3.28 and 4.2 mg kg^− 1^ FW in alkaline and acidic soils, respectively. In contrast to Basmati-198, which exhibited maximum results in alkaline soil, Basmati-515 demonstrated a significant 16% increase in total chlorophyll content specifically in acidic soil (Figure E, F). We also discovered that raising the concentrations of FeSO_4_ in the soil caused a substantial (p < 0.05) rise in the carotenoids content in alkaline as well as acidic soil (Figure G, H).

### Effect of Fe on Fe accumulation in shoot, root, total accumulation in plants and root-to-shoot Fe ratio of *O. sativa* cultivars in soil (pH 7.5 and pH 5.5)

**Fig. 3:**
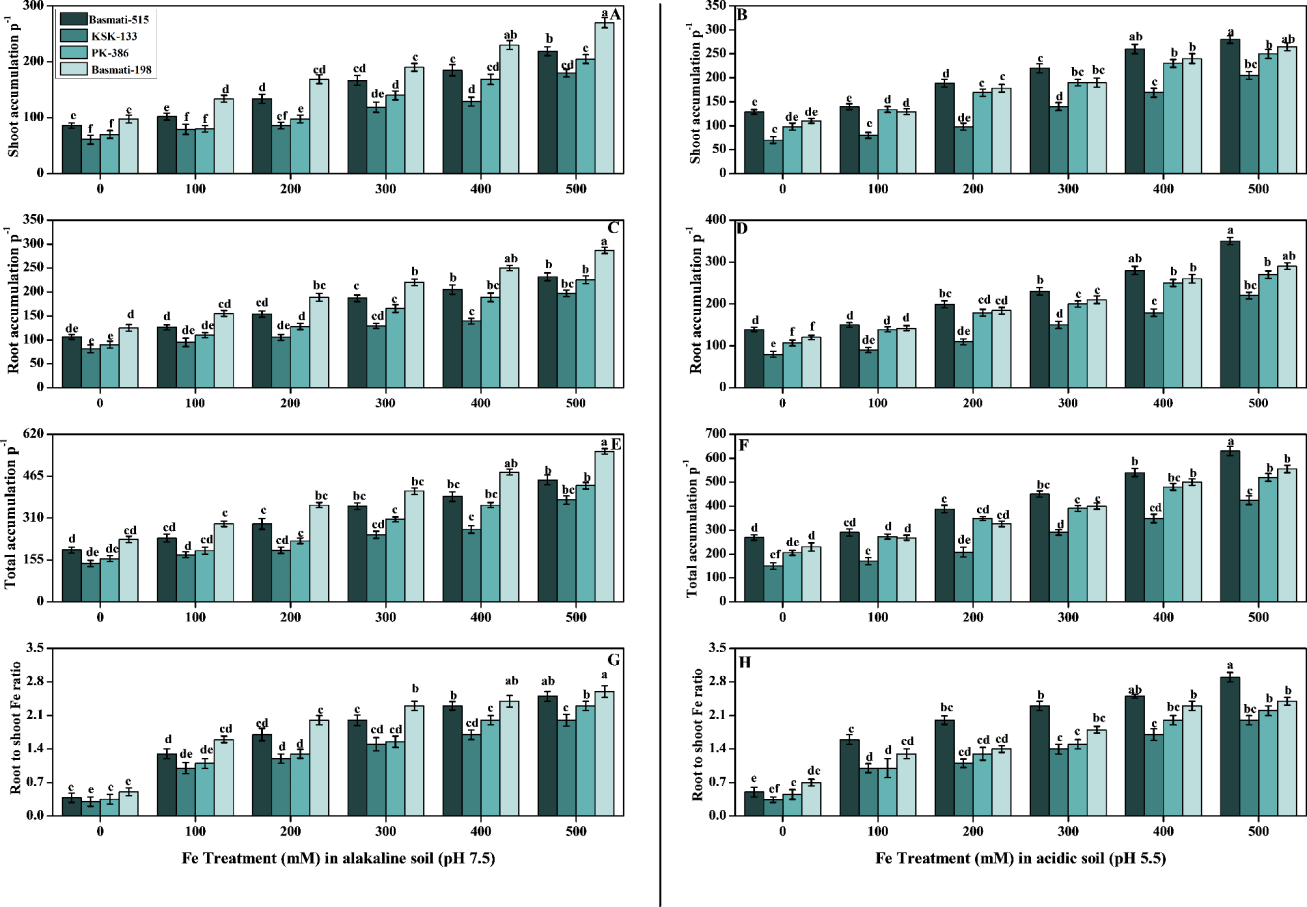
Effect of Fe (0, 100, 200, 300, 400, and 500 mM) on selected *O. sativa* cultivars under alkaline and acidic pH. Fe accumulation in shoot (**A, B**), Fe accumulation in roots (**C, D**), Fe total accumulation in plant (**E, F**), and root to shoot Fe ratio (**G, H**). According to DMRT, bars with distinct alphabet letters are substantially (p < 0.05) different from one another. All data in the graph are the averages of six replicates (n = 6)

We assessed another important effect of application of FeSO_4_ on iron accumulation in shoots, roots, and total Fe accumulation in plants, as well as root-to-shoot Fe ratio (Fig. 3). This showed that by increasing the levels of FeSO4 contents, iron accumulation was also increased. As compared to the control plants, iron accumulation increased in all cultivars of *O. sativa* under the different treatments of Fe. Basmati-198 accumulated the most Fe in alkaline soil, while Basmati-515 accumulated the higher Fe in acidic soil (Fig. 3). The root-to-shoot Fe ratio was also elucidated, with data demonstrating that increasing FeSO_4_ concentrations resulted in a substantial (p < 0.05) rise in Fe contents of *O. sativa* cultivars when compared to control (Fig. 3). Iron accumulation in the shoot of the *O. sativa* cultivar Basmati-198 exhibited a significant increase of 175% in alkaline soil and 117% in acidic soil compared to the respective control conditions (Fig. 3A, B). However, all other parameters, including iron accumulation in the root, total accumulation, and root to shoot ratio, showed a significant positive response towards the highest concentrations of FeSO_4_ in alkaline and acidic soil (Fig. 3C–H). Upon further analysis, it was found that Basmati-515 exhibited the highest iron accumulation in roots when treated with a high concentration of FeSO_4_ (500 mM), with an average value of 350 root accumulation P^− 1^. Notably, Basmati-515 displayed an 18% increase in iron accumulation in acidic soil compared to Basmati-198, which exhibited its highest response in alkaline soil with a value of 287 root accumulation P^− 1^ (Fig. 3C, D). The *O. sativa* cultivar Basmati-198 demonstrated the highest total iron accumulation in alkaline soil, while Basmati-515 displayed a more favourable response in acidic soil, reaching a maximum total accumulation of 630 and 555 total accumulation P^− 1^, respectively at 500 mM. Furthermore, when comparing the two Basmati cultivars, Basmati-515 exhibited a notable 13% increase in total accumulation in acidic soil (Fig. 3E, F). Root to shoot iron accumulation ratio was increased in the same pattern as iron accumulation in leaves, roots, and total accumulation in plants. In terms of root-to-shoot iron accumulation, Basmati-198 exhibited an average value of 2.6 in treated plants in alkaline soil. On the other hand, Basmati-515 displayed a higher average value of 2.9 in acidic soil compared to untreated plants. When comparing the two cultivars, Basmati-515 demonstrated significantly higher results, with an 11% increase in iron accumulation, specifically in acidic soil. This indicates the stronger responsiveness of Basmati-515 to iron treatment in an acidic soil environment. (Fig. 3G, H).

### Effect of Fe on antioxidant enzymatic activity (CAT, APX, POD and SOD) of *O. sativa* cultivars in soil (pH 7.5 and pH 5.5)

**Fig. 4:**
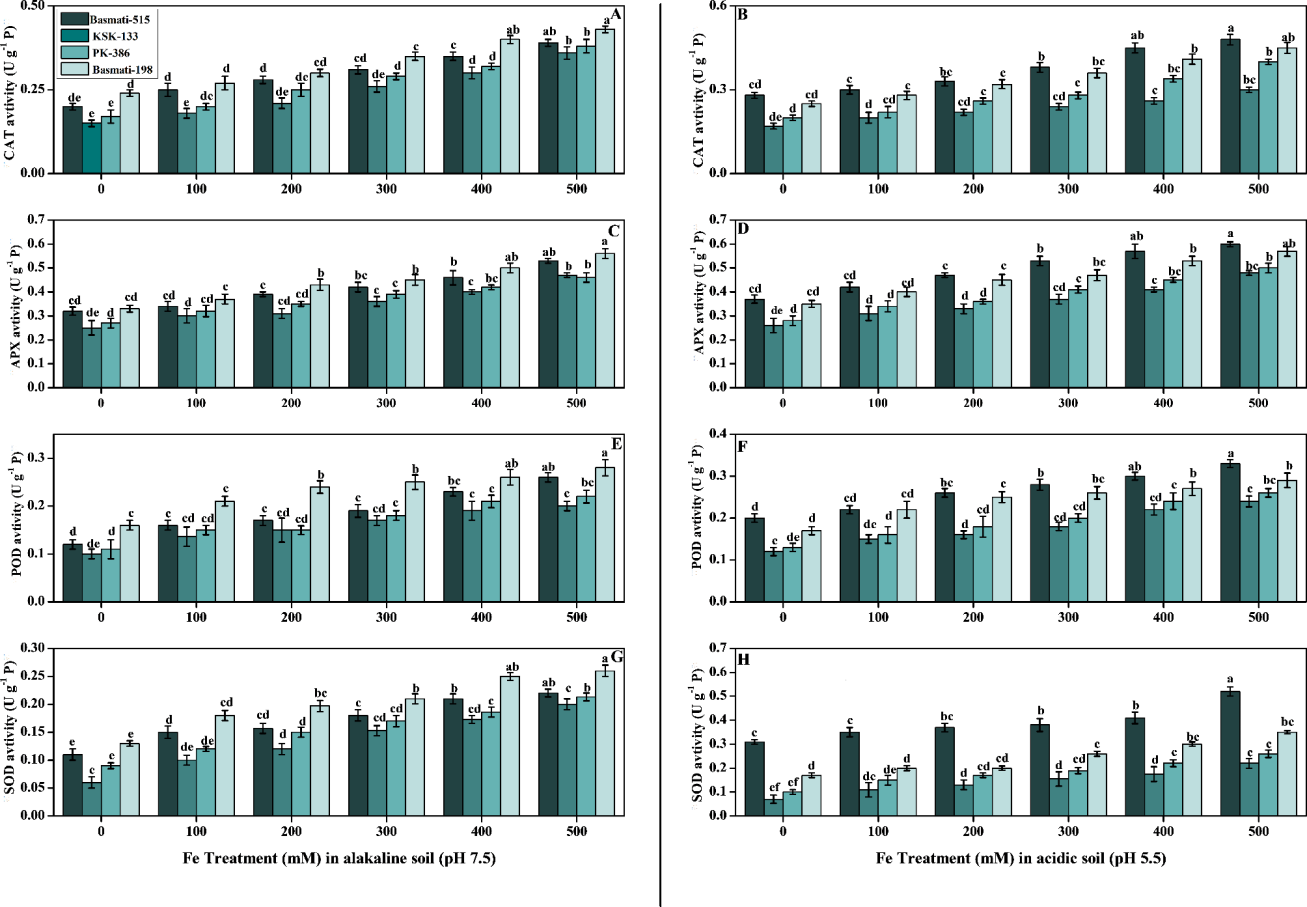
Effect of Fe (0, 100, 200, 300, 400, and 500 mM) on selected *O. sativa* cultivars under alkaline and acidic pH. CAT activity (**A, B**), APX activity (**C, D**), POD activity (**E, F**), and SOD activity (**G, H**). According to DMRT, bars with distinct alphabet letters are substantially (p < 0.05) different from one another. All data in the graph are the averages of six replicates (n = 6)

The data related to the antioxidant enzymatic activity (CAT, APX, POD and SOD) of *O. sativa* cultivars in alkaline (pH 7.5) and acidic soil (pH 5.5) as depicted in Fig. 4. The treatment of different doses of iron has a positive effect on all antioxidant enzymes and by increasing the concentration of FeSO_4_ significantly high results were obtained in Basmati-515. Maximum antioxidant enzymatic activity was observed in Basmati-198 in alkaline soil, while Basmati-515 showed the maximum antioxidants in acidic soil (Fig. 4A-H). A dose of 500 mM FeSO_4_ induced a significant increase in CAT activity in alkaline and acidic soils, with values of 0.43 and 0.48 UgP^− 1^ in Basmati-198 and Basmati-515, respectively. Basmati-198 displayed a notable 69% increase in APX (ascorbate peroxidase) activity in alkaline soil, whereas Basmati-515 exhibited maximum results in acidic soil with a 62% increase in APX activity Fig. 4 (C, D). Basmati-198 demonstrated a significant 55% increase in POD activity in alkaline soil, while Basmati-515 displayed the highest results with a remarkable 65% increase in POD activity specifically in acidic soil (Fig. 4E, F). Basmati-515 showed a maximum SOD value at a level of 400 and 500 mM of FeSO_4_, with an average value of 0.41 and 0.52 UgP^− 1^ higher than the control, which has a value of 0.31 UgP^− 1^. Basmati-515 exhibited a substantial 95% higher increase in acidic soil compared to Basmati-198 (Fig. 4G, H). This highlights the superior responsiveness of Basmati-515 in terms of the observed increase in all antioxidants.

### Effect of Fe on antioxidant enzymatic activity (GPX and DPPH) and oxidative markers (MDA and H_2_O_2_) of *O. sativa* cultivars in soil (pH 7.5 and pH 5.5)

**Fig. 5:**
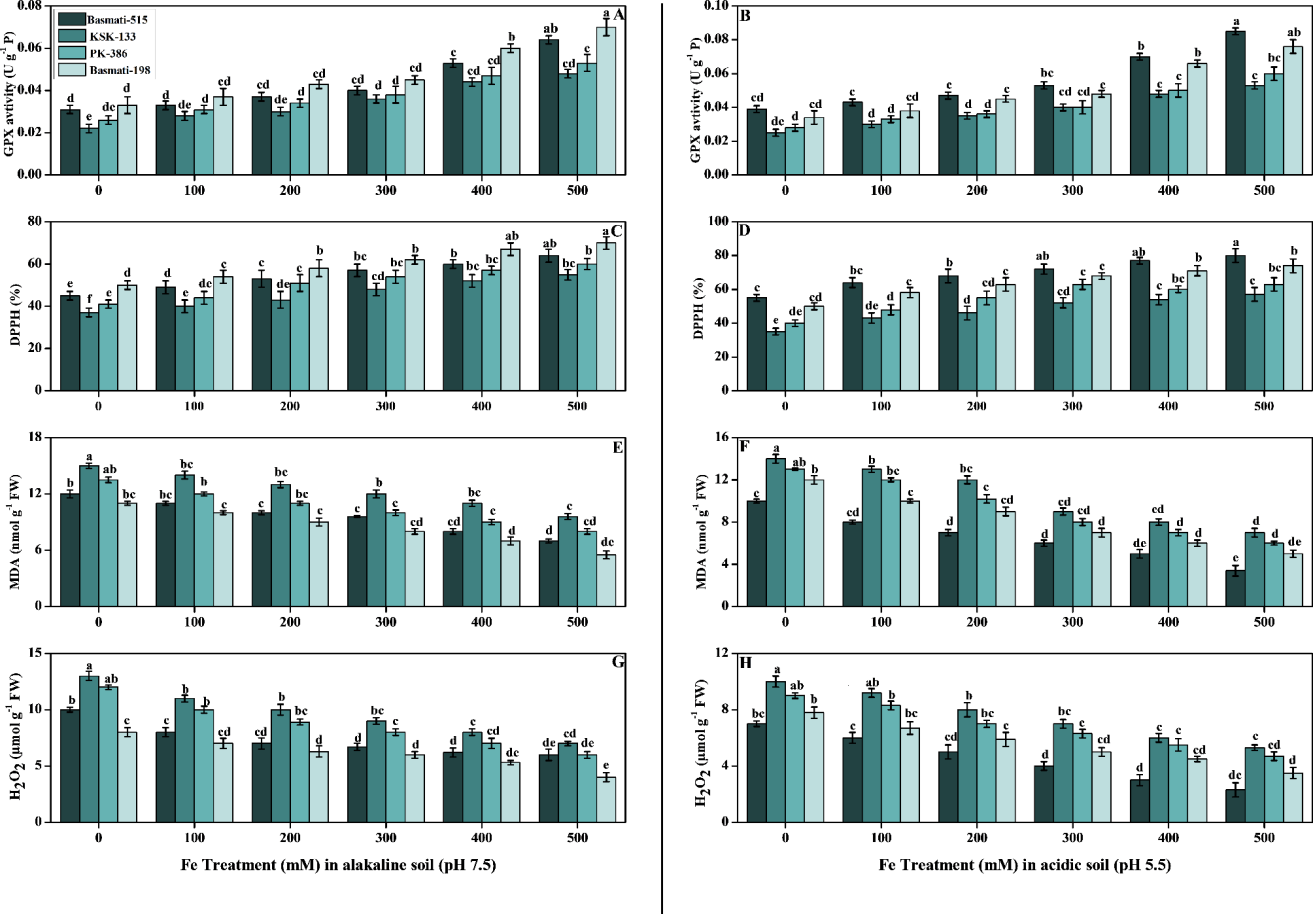
Effect of Fe (0, 100, 200, 300, 400, and 500 mM) on increasing the level of antioxidants (GPX (**A, B**); DPPH (**C, D**)) and reducing oxidative stress levels (MDA (**E, F**); H_2_O_2_ (**G, H**)) in selected *Oryza sativa* cultivars under alkaline and acidic pH. According to DMRT, bars with distinct alphabet letters are substantially (p < 0.05) different from one another. All data in the graph are the averages of six replicates (n = 6)

Figure 5 depicts another important effect of the application of Fe on the antioxidant enzymes and total antioxidant activity (GPX and DPPH), as well as the reduction in oxidative stress markers (MDA and H_2_O_2_) in *O. sativa*. The results showed that, by increasing the application treatment of FeSO_4_, GPX and DPPH increased, as in the case of the other antioxidant enzymes in Fig. 4. As compared to the control plants, GPX and DPPH increased in all cultivars of *O. sativa* under the different treatments of Fe. GPX activity was high in Basmati-198 in alkaline soil, while in acidic soil, Basmati-515 showed a significant high result with a value of 0.07 and 0.085 UgP^− 1^, respectively (Fig. 5A, B). When compared to the control in acidic soil at 80%, increasing the treatment level of FeSO_4_ in the soil resulted in a substantial (p < 0.05) rise in the DPPH of *O. sativa* cultivars (Fig. 5D). In contrast to antioxidants, oxidative stress markers (MDA and H_2_O_2_) decreased in all *O. sativa* cultivars. Basmati-198 exhibited a 50% decrease in MDA (malondialdehyde) content in alkaline soil, while Basmati-515 showed a 40% decrease in MDA in acidic soil. Comparatively, Basmati-515 displayed a 9% greater decrease in MDA in acidic soil compared to Basmati-198. (Fig. 5E, F). In terms of H_2_O_2_ (hydrogen peroxide) content, Basmati-198 demonstrated a 39% decrease in alkaline soil, whereas Basmati-515 exhibited a more substantial 67% decrease in acidic soil when compared to untreated plants. Furthermore, in alkaline soil, Basmati-515 displayed a 54% greater decrease in H_2_O_2_ content compared to Basmati-198 (Fig. 5G, H). The oxidative stress levels were significantly lower in acidic soil compared to all treated and untreated plants in alkaline soil.

### Effect of Fe on soluble sugar, flavonoids, free amino acids, and carbohydrates of *O. sativa* cultivars in soil (pH 7.5 and pH 5.5)

**Fig. 6:**
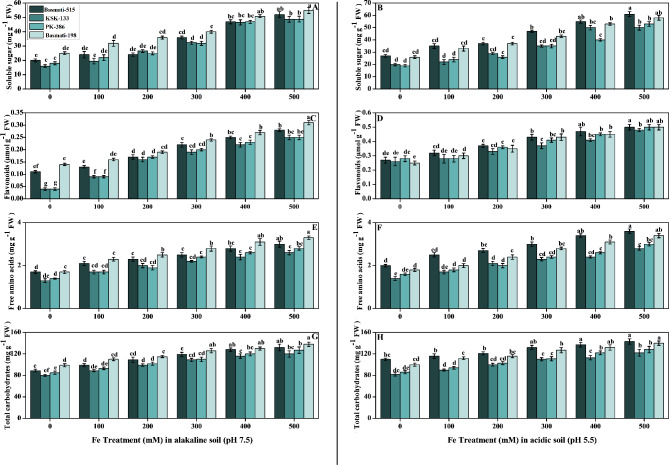
Effect of Fe (0, 100, 200, 300, 400, and 500 mM) on selected *O. sativa* cultivars under alkaline and acidic pH. Soluble sugar (**A, B**), flavonoids (**C, D**), free amino acids (**E, F**), and total carbohydrates (**G, H**). According to DMRT, bars with distinct alphabet letters are substantially (p < 0.05) different from one another. All data in the graph are the averages of six replicates (n = 6)

The effect of application of FeSO_4_ on antioxidants non-enzymatic activities (soluble sugar, flavonoids, total free amino acids, and carbohydrates) in *O. sativa* cultivars is presented in Fig. 6. By increasing the concentration of FeSO_4_, all non-enzymatic activities were increased, in the case of the antioxidant enzymes as compared to the control (Fig. 4). Soluble sugar was significantly higher in Basmati-198 in alkaline soil, while in acidic soil, Basmati-515 showed a significant high result with a value of 48.8 and 61 mg g^− 1^ FW, respectively (Fig. 6A, B). The application of increased FeSO4 concentrations in alkaline soil resulted in a statistically significant improvement in flavonoid content in Basmati-198, compared to the control (0.31 µmol g-1 FW). Notably, the highest flavonoid content was observed in Basmati-515, with an average value of 0.5 µmol g-1 FW. These findings, demonstrate the potential of FeSO_4_ treatment in promoting flavonoid accumulation, with Basmati-515 displaying a better response in terms of flavonoid content (Fig. 6 C, D). Basmati-198 exhibited a substantial 76% increase in free amino acid content, while Basmati-515 displayed an even greater 80% increase. These results indicate the potential of both Basmati-198 and Basmati-515 varieties to enhance the accumulation of free amino acids when treated with FeSO_4_ (Fig. 6E, F). In a comparison between Basmati-515 and Basmati-198, Basmati-515 exhibited a 7% higher increase in carbohydrate content in acidic soil compared to Basmati-198 in alkaline soil. This suggests that Basmati-515 is more responsive to acidic soil conditions in terms of carbohydrate accumulation (Fig. 6G, H).

## Discussion

Iron is required for a variety of metabolic activities in plants. However, its low availability in high pH soils and roots’ reduced ability to acquire it due to iron are two of the most important problems restricting plant development [33]. Micronutrient deficiencies, encompassing calcium, selenium, zinc, and iron, are prevalent among a significant portion of the global population. These deficiencies contribute to malnutrition and pose a substantial burden on public health worldwide [34]. Zinc (Zn) and Iron (Fe) are the two most commonly deficient micronutrients, and they both play vital roles in supporting overall health and well-being [35]. Alkaline soils demonstrate a lack of micronutrients on a global scale [36]. The required levels of micronutrients vary depending on the soil type and pH, necessitating different nutrient considerations for optimal plant growth, and development [37]. pH of the soil is a key component that influences nutrient availability for plants, and soil in arid areas is generally alkaline with a high pH [38]. Despite the widespread recognition of chlorosis occurring in alkaline soils with limited iron (Fe) availability, the antioxidant and physiological responses to iron deficiency remain poorly characterized. There is a lack of comprehensive understanding regarding the specific mechanisms and responses associated with iron deficiency, highlighting the need for further research in this area. Exploring the antioxidant and physiological dynamics under iron-deficient conditions can contribute to a more comprehensive understanding of the impact of iron deficiency on plant health and provide insights into potential strategies for mitigating the detrimental effects of iron deficiency in alkaline soil environments. In this study, we examined the effects of pH and iron (Fe) supply as two factors on plant responses. Our findings revealed that plant responses to Fe supply were significant in acidic soil pH but not in alkaline pH. The experiment involved five Fe treatments at pH levels of 5.5 and 7.5. *O. sativa* cultivars grown in soil treated with FeSO_4_ exhibited enhanced plant growth, improved antioxidant activity, and increased iron accumulation. These results emphasize the importance of considering both soil pH and iron supplementation in optimizing plant performance and nutrient uptake in rice cultivation.

Fe is a crucial micronutrient that plays a significant role in numerous biological processes and is essential for both human health and plant vitality [39]. In alkaline soils with a pH ranging from 7.4 to 8.5, iron minerals tend to have low solubility and slow dissolution kinetics. The uptake of iron by plants growing in alkaline soils is further hindered by elevated bicarbonate levels, a characteristic feature of calcareous soils. As a result, the concentration of available iron becomes inadequate for optimal plant growth, leading to common occurrences of iron deficiency in plants [40]. Fe is necessary for the plants growth, and development. While iron is present in abundance in soil, the fraction of iron that is readily available for plant uptake is often limited. This limited availability of accessible iron in the soil can pose a challenge for plants to meet their iron requirements, potentially leading to iron deficiency [41]. *O. sativa* cultivars grown in soil treated with FeSO_4_ (100, 200, 300, 400 and 500 mM), both in acidic and alkaline soil conditions, exhibited notable improvements in various morphological characteristics, including growth, plant height, and fresh and dry mass. Iron can contribute to increased plant height. It is a crucial component of various enzymes involved in protein synthesis. Proteins are essential for cell division and elongation, which contribute to overall plant growth and height. Adequate iron levels ensure the proper functioning of these enzymes, facilitating optimal protein synthesis and promoting plant height development [42]. Our study, which focused on the impact of FeSO_4_ on plant growth and morphology, is highly relevant to the findings from the previous study regarding the role of iron in plant hormone regulation and cell elongation. The previous study highlighted that iron is involved in the biosynthesis and regulation of plant hormones, specifically auxins. Iron influences the synthesis and distribution of auxins, which, in turn, impacts cell elongation and ultimately contributes to increased plant height [43]. By ensuring sufficient iron availability through FeSO_4_ treatment, our study demonstrated the positive impact of iron on morphological characteristics in *O. sativa* cultivars. The increased iron availability stimulated chlorophyll synthesis, enzymatic activity, and hormone regulation, all of which collectively contributed to improved growth, plant height, and biomass accumulation. The lack of comprehensive studies and understanding regarding the uptake and translocation of iron from the soil has hindered the development of rice cultivars. However, previous research has shown that for cultivars such as BARI-2000 and BARD-699, foliar applications of iron treatments led to growth and photosynthetic rate increases of up to 58% and 70%, respectively. Furthermore, Various morpho-physiological parameters, including shoot length, root length, shoot fresh and dry weights, root fresh and dry weights, photosynthetic and transpiration rates, as well as SPAD values, were also observed to increase [44].

Significant decreases in plant height, shoot fresh weight, root dry weight, germination percentages, and photosynthesis (chl a, chl b, total chlorophyll, and carotenoids content) were observed in iron-deficient rice cultivars in alkaline as well as acidic soil (Figs. 1 and 2). Previous studies that examined morphological traits under different iron levels and chlorophyll contents have shown that iron deficiency can lead to chloroplast degeneration in plant leaves [45]. Salinity refers to high levels of salt in the soil, which can lead to osmotic stress as the high salt levels disrupt water uptake and inhibit nutrient absorption such as iron. Without sufficient iron, the photosynthetic rate of plants can be reduced, leading to decreased shoot and root growth. Additionally, iron deficiency can affect the functioning of stomata, which regulate gas exchange and transpiration rates. Reduced stomatal conductance can further impact photosynthesis and transpiration, limiting plant growth [46, 47]. Iron deficiency can disrupt electron transport and ATP synthesis, leading to reduced energy availability for plant growth and photosynthetic processes [48]. By increasing the levels of FeSO4 contents, iron accumulation was also increased. As compared to the control plants, iron accumulation increased in all cultivars of *O. sativa* under the different treatments of Fe (Fig. 3). FeSO_4_ serves as a source of soluble iron ions (Fe^2+^) that are readily available for uptake by plant roots. As the concentration of FeSO_4_ increases, there is a higher concentration gradient of Fe^2+^ in the soil, which enhances the potential for iron uptake by plant roots, leading to a higher accumulation of iron in the plant tissues [12, 49].

In our current research, we observed significant enhancements in various antioxidants with increased Fe treatment in both alkaline and acidic soil conditions (Fig. 4). These findings highlight the positive impact of FeSO_4_ on multiple biochemical parameters associated with plant health and stress response Specifically, antioxidants such as catalase (CAT), ascorbate peroxidase (APX), peroxidase (POD), superoxide dismutase (SOD), glutathione peroxidase (GPX), and the free radical scavenging capacity measured by the DPPH (2,2-diphenyl-1-picrylhydrazyl) assay exhibited increased activity in response to Fe treatment (Figs. 3 and 4). The positive influence of Fe supplementation on the plant’s antioxidant capacity observed in our research can be related to previous studies that showed the enhancement of antioxidants due to the use of iron oxide nanoparticles [50]. Furthermore, Fe plays a role in regulating the expression of genes involved in antioxidant defense. Fe availability affects the transcription and translation of genes encoding antioxidant enzymes, thereby increasing their production and activity. This ultimately leads to an enhanced antioxidant capacity in Fe-supplemented plants [51]. The reduction in SOD, POD, and CAT activities in *O. sativa* cultivars under significant Fe deficiency. Additionally, our study clarified that, under conditions of significant iron (Fe) deficiency, the activities of key antioxidant enzymes, reduced *O. sativa* cultivars reflects the impact of Fe deficiency on the antioxidant defense system and increase in oxidative stress (Fig. 5). Our study is in line with the findings of previous research that investigated the exogenous application of FeSO_4_ on *O. sativa* plants and its impact on Cd toxicity, antioxidant potential, plant growth, and photosynthesis. The previous study reported that the application of FeSO_4_ resulted in decreased Cd toxicity by enhancing the antioxidant potential and decreasing the MDA and H_2_O_2_ level [52–54].

Furthermore, Fe treatment led to elevated levels of proline, free amino acids, soluble sugars (including reducing and non-reducing sugars), flavonoids, total soluble protein, phenolic compounds, and total carbohydrates (Fig. 6). These biochemical components are involved in various physiological processes, such as osmotic adjustment, stress tolerance, defense mechanisms, and energy metabolism. By establishing a connection between our research and previous studies, it is reported that Fe is known to interact with other components of the antioxidant system, such as non-enzymatic antioxidants like phenolic compounds and flavonoids, further enhancing the antioxidant capacity of the plant [55]. According to our study, increasing the iron concentration level in the soil can lead to an increase in free amino acids and carbohydrates and enhance the uptake of iron by plants. These findings are in agreement with previous studies. Iron levels lead to increased carbohydrate production in plants, strengthen cell walls, and provide building blocks for the synthesis of defense-related compounds [56]. Another study showed that iron is essential for maintaining optimal amino acid metabolism in plants [57, 58]. A significant increase in flavonoid content in *O. sativa* cultivars was caused by increased FeSO_4_ concentrations in comparison to the control (0.27 µmol g^− 1^ FW) in acidic soil at 500 mM (Fig. 6D). In addition to acting as antioxidants, flavonoids also contribute to plant metal tolerance by donating hydrogen atoms. It has been demonstrated that plants are more tolerant of stress when their flavonoids are higher [59].

**Fig. 7:**
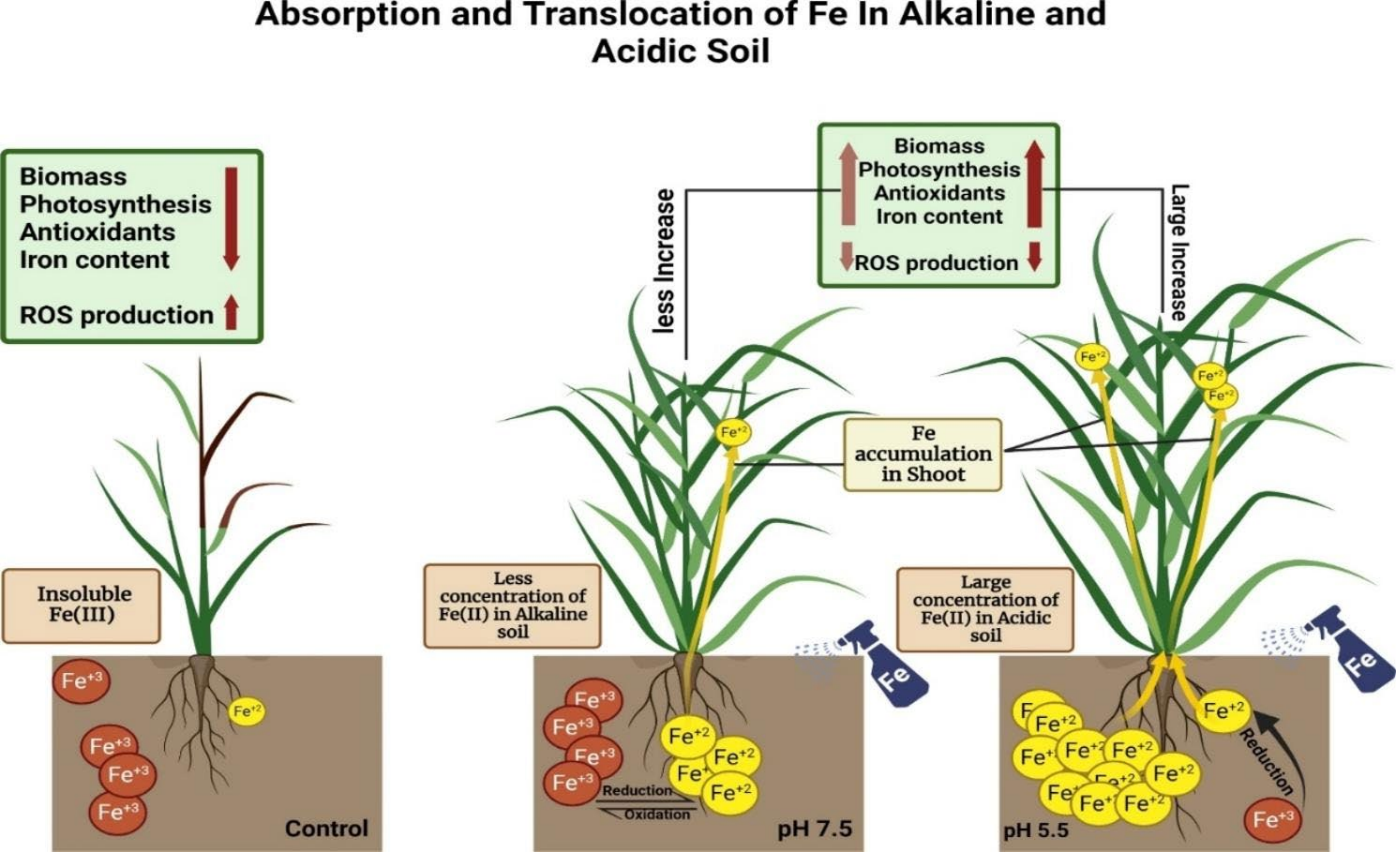
Impact of iron fertilizer treatment on iron accumulation in rice plants and pH-dependent solubility

It was observed in the present study that iron availability in the soil is highly influenced by soil pH. In alkaline soils, iron tends to form insoluble compounds, making it less available for plant uptake. However, Fe treatment enables them to better access and acquire iron in alkaline soil. On the other hand, in acidic soils, iron is generally more soluble and available for plant uptake; therefore, *O. sativa* cultivars adapted to acidic soil conditions under Fe treatment efficiently take up and accumulate iron. Furthermore, the efficiency of iron uptake and transport is closely related to the characteristics of the plant’s root system, morphology, and antioxidants (Fig. 7). Different cultivars, such as Basmati-515 and Basmati-198, exhibited variations in morphology and antioxidants that influenced the cultivar’s ability to acquire iron from the soil, allowing them to accumulate higher iron levels in either alkaline or acidic soil conditions. Further research is needed to explore the genetic and physiological basis for the differential iron accumulation observed in various cultivars and soil pH conditions.

## Conclusion

On the basis of current findings, it can be concluded that Fe deficiency caused a significant decrease in all morphological characteristics such as plant height, root and shoot fresh and dry biomass, total plant biomass production, photosynthetic activity, and gas parameters due to the lower availability of Fe in alkaline soil. While significant increases in oxidative stress markers (MDA and H_2_O_2_) increased in Fe deficient conditions, which were reduced by treatment with FeSO_4_ by the production of CAT, APX, POD, SOD, GPX and DPPH. Thus, the application of Fe is a safer and better way to increase iron content in *O. sativa* as well as biomass production. Basmati-515 and Basmati-198 performed better in alkaline and acidic soils, respectively, than the control and other cultivars of *O. sativa*. However, the acidic and alkaline restrictions on iron availability in soil, as well as the influence of pH on iron transporter genes, must be investigated further at the molecular level.

## Data Availability

The datasets used and/or analyzed during the current study are available from the corresponding author on reasonable request.
